# Cerebral cortex and blood transcriptome changes in mouse neonates prenatally exposed to air pollution particulate matter

**DOI:** 10.1186/s11689-021-09380-3

**Published:** 2021-08-24

**Authors:** Amin Haghani, Jason I. Feinberg, Kristy C. Lewis, Christine Ladd-Acosta, Richard G. Johnson, Andrew E. Jaffe, Constantinos Sioutas, Caleb E. Finch, Daniel B. Campbell, Todd E. Morgan, Heather E. Volk

**Affiliations:** 1 Leonard Davis School of Gerontology, University of Southern California, Los Angeles, CA USA; 2 Department of Human Genetics, David Geffen School of Medicine, University of California Los Angeles, Los Angeles, CA USA; 3 Department of Mental Health, Johns Hopkins Bloomberg School of Public Health, Baltimore, MD USA; 4 Wendy Klag Center for Autism and Developmental Disabilities, Johns Hopkins Bloomberg School of Public Health, Baltimore, MD USA; 5 Department of Pediatrics and Human Development, Michigan State University, Grand Rapids, MI USA; 6 Department of Epidemiology, Johns Hopkins Bloomberg School of Public Health, Baltimore, MD USA; 7 Lieber Institute for Brain Development, Baltimore, MD USA; 8 Department of Biostatistics, Johns Hopkins Bloomberg School of Public Health, Baltimore, MD USA; 9 Center for Computational Biology, Johns Hopkins University, Baltimore, MD USA; 10 Lieber Institute for Brain Development, Johns Hopkins Medical Campus, Baltimore, MD USA; 11 Department of Civil and Environmental Engineering, Viterbi School of Engineering, University of Southern California, Los Angeles, CA USA; 12 Department of Environmental Health and Engineering, Johns Hopkins Bloomberg School of Public Health, Baltimore, MD USA

**Keywords:** nPM, RNA sequencing, Cerebral cortex, Blood

## Abstract

**Background:**

Prenatal exposure to air pollutants is associated with increased risk for neurodevelopmental and neurodegenerative disorders. However, few studies have identified transcriptional changes related to air pollutant exposure.

**Methods:**

RNA sequencing was used to examine transcriptomic changes in blood and cerebral cortex of three male and three female mouse neonates prenatally exposed to traffic-related nano-sized particulate matter (nPM) compared to three male and three female mouse neonates prenatally exposed to control filter air.

**Results:**

We identified 19 nPM-associated differentially expressed genes (nPM-DEGs) in blood and 124 nPM-DEGs in cerebral cortex. The cerebral cortex transcriptional responses to nPM suggested neuroinflammation involvement, including CREB1, BDNF, and IFNγ genes. Both blood and brain tissues showed nPM transcriptional changes related to DNA damage, oxidative stress, and immune responses. Three blood nPM-DEGs showed a canonical correlation of 0.98 with 14 nPM-DEGS in the cerebral cortex, suggesting a convergence of gene expression changes in blood and cerebral cortex. Exploratory sex-stratified analyses suggested a higher number of nPM-DEGs in female cerebral cortex than male cerebral cortex. The sex-stratified analyses identified 2 nPM-DEGs (Rgl2 and Gm37534) shared between blood and cerebral cortex in a sex-dependent manner.

**Conclusions:**

Our findings suggest that prenatal nPM exposure induces transcriptional changes in the cerebral cortex, some of which are also observed in blood. Further research is needed to replicate nPM-induced transcriptional changes with additional biologically relevant time points for brain development.

**Supplementary Information:**

The online version contains supplementary material available at 10.1186/s11689-021-09380-3.

## Background

Poor air quality remains a leading global risk factor of mortality and disability in humans [1]. The gestational period is one of the most vulnerable life-stages for air pollution exposure, with potential long-term impacts on human health [2]. Several meta-analyses have shown that prenatal air pollution exposure is associated with premature birth [3], low birth weight [4], and other longer-term health outcomes including cardiovascular disease [5, 6], elevated blood pressure [7, 8], and childhood cancer [9, 10]. A growing body of research further indicates that prenatal air pollution exposure, and particulate matter (PM) specifically, may affect brain processes throughout life. Prenatal exposure to PM has been associated with an increased risk of autism spectrum disorder (ASD) [11–15], childhood hyperactivity [16, 17], and cognitive impairments [18–20]. Moreover, there is a dose-response relationship between prenatal PAH exposure and subsequent reductions in brain white matter surface, attention deficits, and hyperactivity in childhood [17, 21, 22]. Numerous epidemiological studies have reported sex-specific toxicity of prenatal air pollution exposure [23–26]. For example, while boys show increased air pollution-mediated cognitive decline in attention domains [24, 27], girls show more vulnerability in memory domains [24]. ASD is also diagnosed 4 times more often in males than in females, and air pollution exposure is a major contributor to ASD risk [11–15].

Studies from animal models similarly show effects of air pollution on the developing brain. Prenatal exposure to urban particulate matter (PM2.5 or PM0.2) caused impaired neurogenesis, blood-brain barrier leakage, hippocampal mitochondrial damage, ventriculomegaly, and neuroinflammation during adulthood [28–32]. These changes were accompanied by spatial memory deficits and depressive behaviors [28, 30]. Several developmental effects of prenatal air pollution exposure have been reported, including depressive behaviors [30], excess body weight [31], neurogenesis decline [33], hypermyelination [34], neuroinflammation [35], microgliosis [35], and astrogliosis [36].

Despite the animal model and observational epidemiology associations, there is a lack of information about molecular changes in brain tissue in response to prenatal air pollutant exposure. Prior microarray analysis of frontal cortex tissue autopsies from children and young adults with different air pollution exposure levels identified around 134 differentially expressed genes; the genes were predominantly part of inflammation and antioxidant response pathways [37]. Because air pollution data in humans is not controlled, rodent models have been used to define brain transcriptome changes related to prenatal air pollutant exposure. Similar to human studies, changes in inflammatory genes have been identified. For example, in a study of adult rat brains, chronic exposure to PM0.2 changed the expression of some genes related to inflammation, calcium channels, and glutamate receptors [38]. Chronic diesel exhaust inhalation caused gene expression changes related to inflammation of adult mice olfactory bulbs [39]. However, there is a lack of assessment of gene expression changes in neonates in both brain and blood following gestational air pollution exposure. Here, we seek to identify transcriptome changes that occur in a rodent model, across both brain and blood tissues of the neonates, following prenatal exposure to nano-size particulate matter (nPM). The siblings of these animals showed chronic weight and fat gain, plus a male-specific depressive behavior and glucose intolerance [40]. Thus, the current study focuses on the neonate siblings to identify the initial brain and blood responses that can be used as biomarkers or therapeutic targets. Moreover, this study will inform interpretation and comparison of results across rodent and human studies where only peripheral blood tissues are accessible. For the first time, we aim to identify initial transcriptome responses to prenatal air pollution exposure in early developmental stages.

## Methods

### Animals and ethics statements

Male and female C57BL/6NJ mice were purchased from Jackson Laboratory. The experimental protocols were approved by the University of Southern California Institutional Animal Care and Use Committee (protocol #11992 and 20720). The study followed the recommendations in the Guide for the Care and Use of Laboratory Animals of the National Institutes of Health.

### Particulate matter collection

nPM is a nano-sized (diameter < 200 nm) particulate matter sub-fraction of ambient air pollution collected near CA-110 Freeway in Los Angeles following prior protocols [41]. Briefly, nPM was collected on Teflon filters and resuspended in water using sonication. For animal exposure, the suspended nPM was re-aerosolized at 300 μg/m^3^ concentration. While this dose is relatively high, the 3 weeks of intermittent exposure during pregnancy (5 h/day, 3 days/week) to 300 μg/m^3^ nPM yields an average hourly exposure of 27 μg/m^3^, as experienced in many cities [42]. The nPM composition and size distribution are characterized in our previous studies [41, 43, 44].

### Gestational exposure

Nine-week-old C57BL/6NJ mice were obtained from Jackson Laboratory. Females were housed together for a week to suppress the ovulation cycle due to the Whitten Effect. Mice were weighed, and ear-tagged for identification. Shavings from the male’s cage were introduced 3 days before the formation of the breeding pairs to restart the ovulation cycle. Breeding trios were formed by placing two females and one male into a fresh cage just before the dark cycle. Males were then removed 3 days later; females remained paired. Five breeding trios, each producing viable offspring in the initial co-housing, were randomly assigned into two treatment groups: nPM and filter air-exposed. The day after observing a copulatory plug, mice were exposed to re-aerosolized nPM (300 μg/m^3^) for 5 h/day, 3 days/week from 10:00 to 15:00 each day. The standard housing cages containing the mice were placed into large exposure chambers that were identical for nPM and filtered-air. Temperature and airflow were controlled for adequate ventilation and to minimize the buildup of animal-generated contaminants [skin dander, carbon dioxide (CO2), ammonia]. Exposure stopped with the birth of the first pup. The five breeding trios per treatment group had viable litters with 35 neonates for nPM and 33 for filter-air exposed. Prior to euthanasia and sample collection, the sex of the neonates was determined by the distance of the genital papilla and the anal opening. Sex was also confirmed by PCR (see below). Three neonates per sex per exposure group from different litters were randomly chosen and euthanized at day 5 age for collection of whole blood and dissected cerebral cortex. Samples were snap-frozen and stored at – 80 °C.

### RNA extraction and determination of sex by qPCR

Samples were homogenized and disrupted with pestles, 22-gauge needles, pipetting and QIA shredders (Qiagen #79654). DNA was extracted using QIAzol (Qiagen #79306) from the organic layer for genotyping. RNA was extracted following the QIAGEN RNeasy plus universal protocol (Qiagen #73404). Extracted RNA was used to determine sex of mouse neonates by qPCR for the Y-chromosome *Sry* gene. Following the determination of sex, three males and females per group were randomly selected for further RNA sequencing.

### Library preparation and RNA sequencing of blood and cerebral cortex

RNA (600 ng) was made into RNA-seq libraries using the Illumina RiboZero Gold library preparation kit and sequenced on an Illumina HiSeq 3000 sequencer at the Lieber Institute for Brain Development.

### Preprocessing of the RNAseq data

Raw sequencing reads were quality checked with FastQC (Babraham Bioinformatics, 2016) and, where needed, adapter sequences were trimmed from the reads using Trimmomatic [45]. For all samples, raw sequencing reads ranged between 10 and 30 million reads. Reads were aligned to the mm10 genome using the HISAT2 splice-aware aligner [46] and alignments overlapping genes were counted using featureCounts version 1.5.0-p3 [47] relative to Gencode version M11 (118,925 transcripts across 48,709 genes, March 2016).

Following alignment and mapping of the sequences to the mouse genome, the data was converted to Count per Million (CPM) for data visualization and preliminary assessment using EdgeR package in R. The CPM values were normalized using TMM method (weighted trimmed mean of the log expression ratios) [48]. One of the blood samples was excluded from the analysis due to distinct count distribution compared to others. The genes were further filtered for blood and cortex separately by omitting the duplicates and genes with zero CPM for any sample. The data was next converted to Log2 expression using Voom package in R for further linear modeling.

### Differential expression analysis of blood and cerebral cortex based on nPM exposed and non-exposed groups

The expression differences of each gene were calculated using Empirical Bayes Statistics (eBayes) in the Limma package [49]. In the large model, the nPM effect was studied after adjustment for sex as a co-variate. We assessed statistical significance using a false discovery rate (FDR) of 5% (*q* value < 0.05) [50]. We set a nominal significant level at *p* value < 0.005, where no genes was detected at *q* < 0.05. This nominal *p* value was selected to get a sufficient number of DEGs that can be used for enrichment analysis; however, we acknowledge that our statistical power is limited by sample size, and the findings need to be validated by a higher sample size in future studies.

### Sex-stratified differential expression analysis of nPM effects in blood and cerebral cortex

The analysis was done as described before but in the sex-stratified data. The overlapped differentially expressed genes (DEGs) were identified between blood and cerebral cortex of male and female neonates.

### Ingenuity pathway analysis of identified gene sets

The identified genes were further studies by Ingenuity Pathway Analysis (IPA) software. The enriched canonical pathways and candidate upstream regulators were calculated based on right-tailed Fisher’s exact test of the overlap of observed genes with the database. Enrichment of the diseases and bio-functions was done by calculating *z*-scores based on the direction of the observed expression profile. *Z*-score is a statistical measure that compares the direction of observed changes to the expression signature of a specific disease constructed from prior kinds of literature.

### Comparison of sex-specific nPM effects across blood and cerebral cortex

We used two approaches to identify the potential blood marker for the air pollution-mediated transcriptome changes in the cerebral cortex. Linear modeling identified shared nPM responses between cerebral cortex and blood in stratified analysis. Sparse canonical correlation analysis using penalized matrix decomposition (sparse CCA) selected blood genes with maximum canonical correlation with the selected genes in the cerebral cortex [51]. This analysis was done using PMA package in R.

## Results

### Transcriptome changes in blood and cerebral cortex of neonates prenatally exposed to air pollutants

**Fig. 1:**
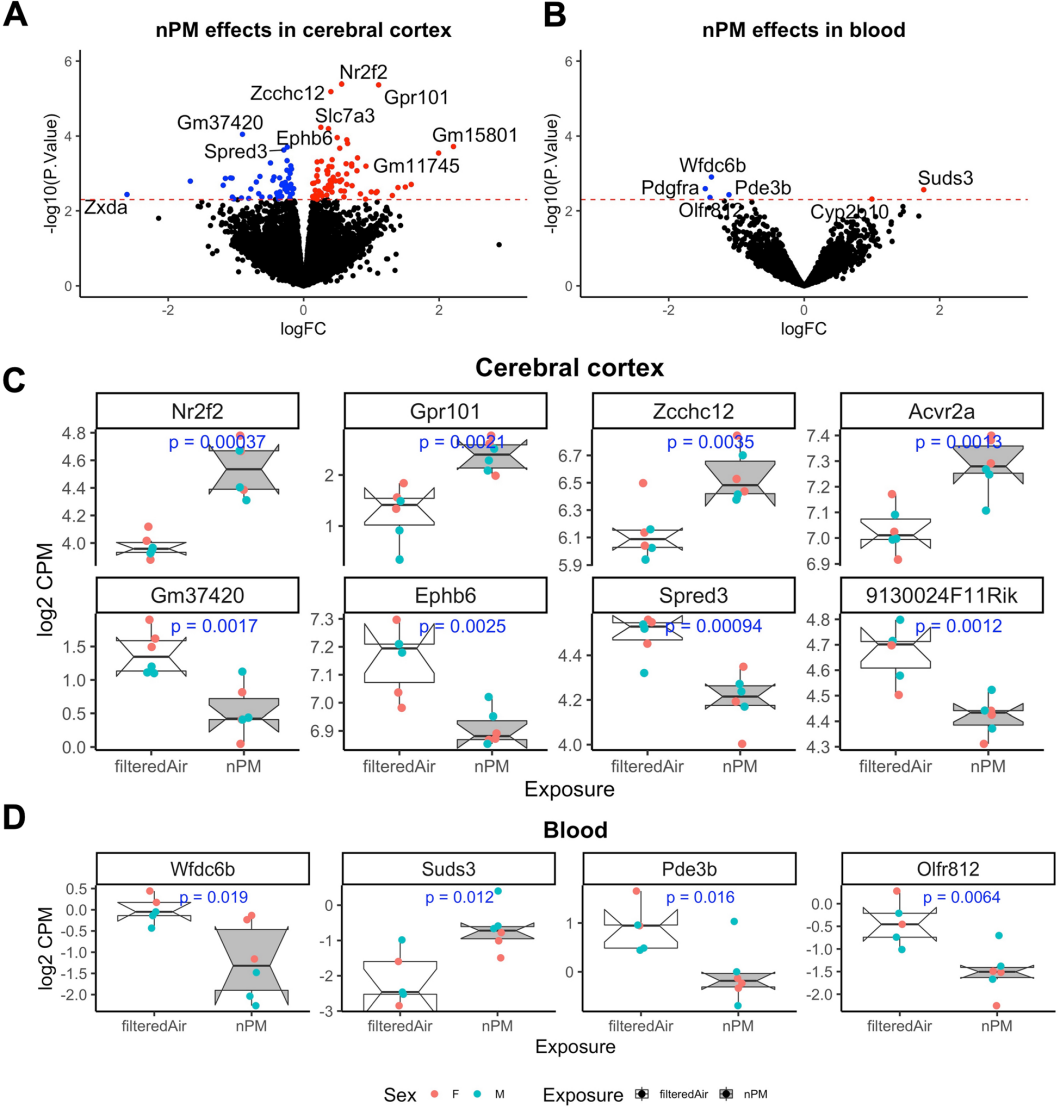
Prenatal exposure of mice to nPM caused a modest gene expression change in the cerebral cortex and blood of neonates. **A** Volcano plot of the cerebral cortex transcriptional changes. The dashed line indicates *p* < 0.005. **B** Volcano plot of the blood transcriptional changes. **C** The top genes that responded to nPM in the cerebral cortex of neonates. **D** The top genes responded to nPM in the blood of neonates. The nPM associations is adjusted for sex and RIN values as co-variates

**Fig. 2:**
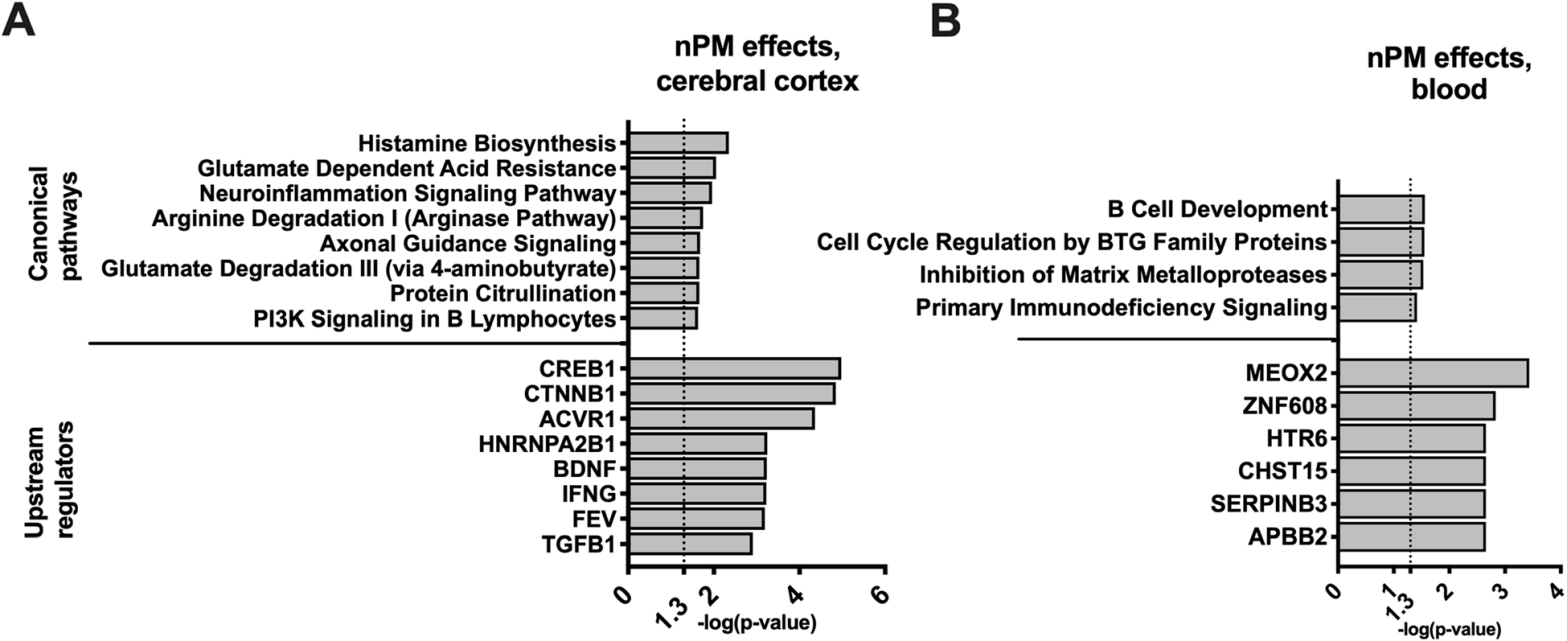
Ingenuity pathway analysis of nPM-associated genes. **A** Canonical pathways and potential upstream regulators of 124 nPM-DEGs in cerebral cortex. **B** Canonical pathways and potential upstream regulators of 19 nPM-DEGs in blood. The DEGs were selected at *p* < 0.005 significance level. The models were adjusted for sex and RIN quality of the input RNA

The total number of detected transcripts in cerebral cortex and blood of the neonates was around 21670 and 10601, respectively. The effects of nPM on blood and cerebral cortex transcriptome were examined by two linear models (Table 1). Using a *p* < 0.005 cutoff, 124 nPM-induced differentially expressed genes (nPM-DEGs) were detected in cerebral cortex and 19 nPM-DEGs were detected in blood (Fig. 1, Table 1). Some of the top genes related to nPM in the cerebral cortex included *Nr2f2*, *Gpr101*, and *Ephb6* (Fig. 1A, C). Ingenuity pathway analysis (IPA) of the 124 nPM-DEGs in cerebral cortex revealed enrichment of genes involved in immune responses (e.g., neuroinflammation and PI3K signaling), neurodevelopment (e.g., axonal guidance), and some metabolic pathways (e.g., glutamate, arginine, and histamine metabolism) (Fig. 2A). The upstream regulators of these nPM-DEGs included cyclic-AMP response element-binding protein (CREB), brain-derived neurotrophic factor (BDNF), and interferon gamma (IFNγ). In blood, only 19 genes responded to prenatal nPM exposure. Some example genes included *Wfdc6b* and *Suds3* (Fig. 1B, D). IPA revealed that the top canonical pathways of the blood nPM-DEGs included immune responses (e.g., B cell development, immunodeficiency signaling), cell cycle regulation, and inhibition of matrix metalloproteases (Fig. 2B). The top upstream regulator of blood nPM-DEGs was mesenchyme homeobox 2 (MEOX2), which regulates vertebrate limb myogenesis, and is also involved in neurovascular dysfunction in Alzheimer's disease.

**Table 1:** Number of DEGs in cerebral cortex and blood of neonates prenatally exposed to nPM

Factors		Cerebral cortex				Blood			
		Model 1		Model 2		Model 1		Model 2	
		*q* value < 0.05	*p* value < 0.005	*q* value < 0.05	*p* value < 0.005	*q* value < 0.05	*p* value < 0.005	*q* value < 0.05	*p* value < 0.005
**nPM vs control**	Up Down	3 0	73 51	0 1	140 242	0 0	7 12	0 1	48 60
**Sex (male vs female)**	Up Down	3 4	38 71	18 30	299 273	0 0	11 16	0 1	41 37
**nPM:sex interaction**				0	267			0	106

Note: The models were adjusted for RNA integrity number (RIN). The models are multivariate linear regression analysis of log2 gene expression. Covariates in model 1: nPM, sex; model 2: nPM, sex, nPM-sex interaction

### Exploratory sex-specific transcriptome changes in blood and cerebral cortex of neonates prenatally exposed to nPM

Since the analysis of the siblings of these animals showed a sex-specific effect of the prenatal nPM exposure in adult mice [40], we explored sex-stratified differences in nPM responses at neonatal stages. Despite the limited sample size, these exploratory analyses could encourage further studies of sex-specific prenatal responses to air pollution. In the following sections, we describe the nPM responses stratified by sexes, but we emphasize that these findings are exploratory and require corroboration by a larger size sample experiment.

As the first approach, a nPM-sex interaction term was included in the model to identify potential differences in transcriptome response to exposure by sex. Interestingly, adding a nPM-Sex interaction term increased the number of DEGs to 382 in cerebral cortex and 108 in blood (Table 1), which suggested the potential sex-specific responses of nPM. Thus, the data were stratified by sex for further downstream analysis.

**Fig. 3:**
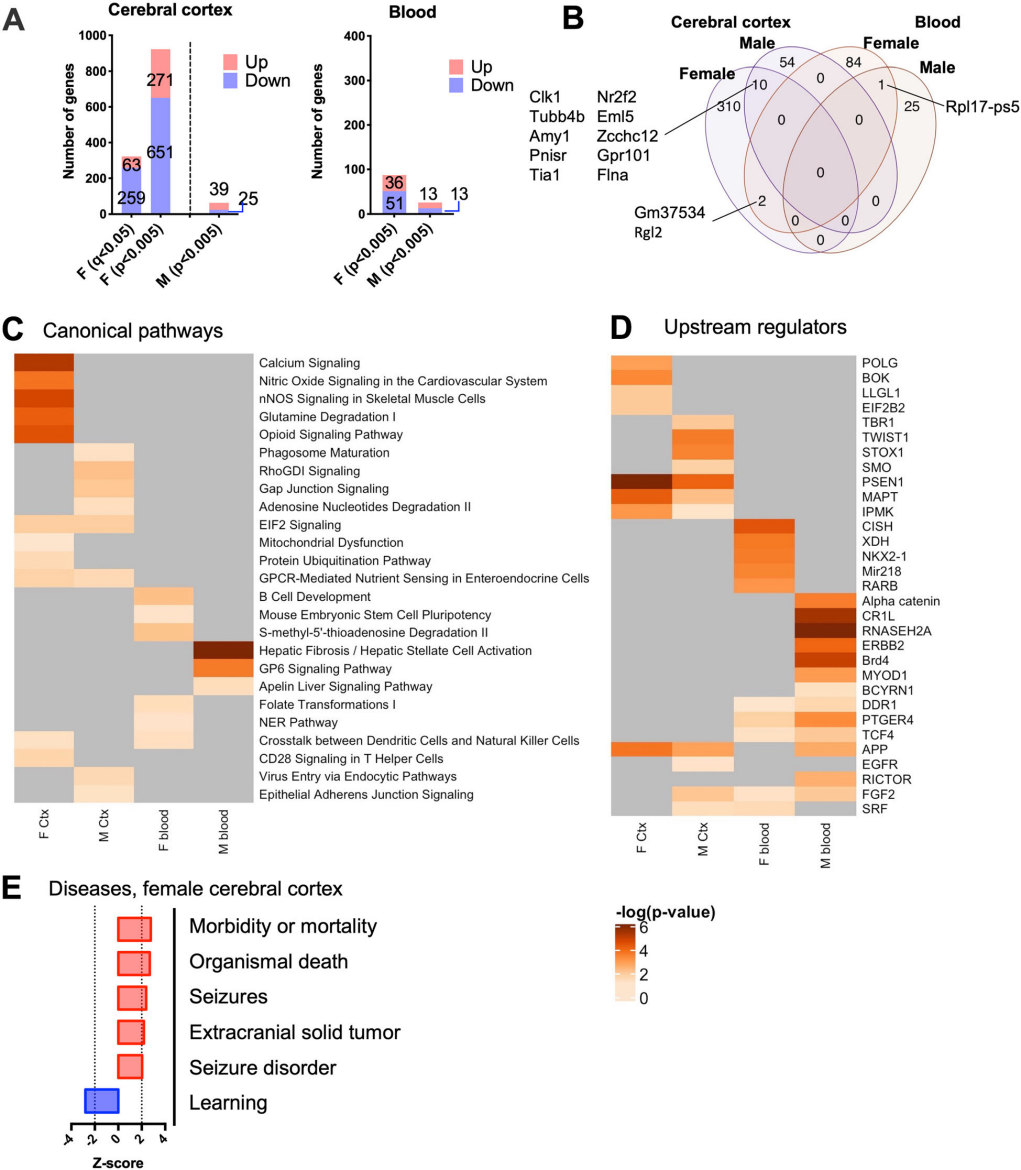
Prenatal exposure of mice to nPM potentially caused sex and tissue-specific gene expression changes in male and female neonates. **A** Differential expression analysis of the cerebral cortex and blood transcriptome responses to nPM. Only female cerebral cortex had DEGs at *q* < 0.05 significance. **B** Venn diagram showing the overlapped DEGs between brain and blood of male and female neonates. For female cerebral cortex, only nPM-DEGs with *q* < 0.05 significance were included in the analysis. **C** Comparison analysis of enriched canonical pathways in all groups. The heatmap shows the top pathways shared between blood and cerebral cortex. **D** Potential upstream regulators of nPM responses in blood and cerebral cortex. The heatmaps are sorted based on the sum of −log10(*p* values) in each row. *P* values below 10^−6^ were converted to 10^−6^ for better visualization. **E** Enriched diseases in the cerebral cortex of females that were prenatally exposed to nPM. *Z*-score is a statistical measure that matches between expected relationship direction built from previous studies and observed gene expression. *Z*-scores > 2 or < − 2 is considered as significant. Note: for female cerebral cortex, only nPM-DEGs with *q* < 0.05 significance were included in the analysis

In the cerebral cortex, only females had 322 nPM-DEGs at 5% FDR (Fig. 3A). The majority of genes (83%) were downregulated following nPM exposure (259 vs. 63). At *p* < 0.005, females had 14-fold more nPM-DEGs than males (922 vs 64). Blood gene responses were lower compared to the cerebral cortex in both male and females. Only one nPM-DEG survived correction for multiple comparisons (Gm23444 gene in females). At *p* < 0.005 significance, 87 nPM-DEGs were detected in female and 26 in male blood samples (Fig. 3A). In both tissues, females had more nPM-DEGs compared to males, which suggests sex-specific gene expression changes. Only 2.6% of changes (10 nPM-DEGs) were shared between male and female cerebral cortex (Fig. 3B). Blood and cerebral cortex only shared two nPM-DEGs in females (Gm37532, and Rgl2).

### Ingenuity pathway analysis of the exploratory sex-specific transcriptional changes in blood and cerebral cortex of neonates prenatally exposed to nPM

IPA was performed on the nPM-DEGs in males and females cerebral cortex and blood. The results of different gene subsets in each group were put together to identify the shared, tissue-, and sex-specific canonical pathways, and potential upstream regulators of the observed changes. The nPM-DEGs in the cerebral cortex were enriched for genes involved in EI2F signaling and GPCR-mediated nutrient sensing pathways (Fig. 3C). In females, nPM-DEGs were enriched for nervous system pathways such as calcium and opioid signaling. Further, nPM-DEGs suggested altered nitric oxide synthesis in the female cerebral cortex. In contrast, nPM-DEFs in males were enriched for pathways such as Gap junction and immune responses (i.e., phagosome maturation, RhoGDI, and CD40 signaling). In blood, nPM-DEGs were enriched for stress and inflammatory-related pathways in both males and females (i.e., G2/M DNA damage checkpoint, p38 MAPK signaling). Some of the sex-specific blood pathways included B cell development, and stem cell pluripotency in females; hepatic fibrosis, and GP6 signaling in males. The shared canonical pathways between blood and cerebral cortex of male or female neonates were associated immune responses (i.e., cross-talk of dendritic and NK cells).

Several potential upstream regulators were enriched in cerebral cortex and blood of the prenatally exposed animals (Fig. 3D). Some of the cerebral cortex regulators included Psen1, and Mapt (Tau) which are associated with Alzheimer disease (AD). The blood-specific upstream regulators of nPM responses included Myod1 (myogenic differentiation 1, related to muscle regeneration), Bcyrn1 (Brain cytoplasmic RNA 1), and Ddr1 (Discoidin domain receptor tyrosine kinase 1, involved in cell growth, differentiation and metabolism). The shared regulators between blood and cerebral cortex included App (amyloid precursor protein, a known AD-associated gene), and Fgf2 (fibroblast growth factor 2).

We also compared the observed nPM-DEGs to gene expression signatures of diseases in the IPA database. Only the changes in the female cerebral cortex could significantly enrich the diseases in IPA. The results suggested that nPM would increase the risk of morbidity, mortality, seizure, tumor formation, and learning impairments in females (Fig. 3E).

### Blood nPM responses as a biomarker of cerebral cortex gene expression changes

**Fig. 4:**
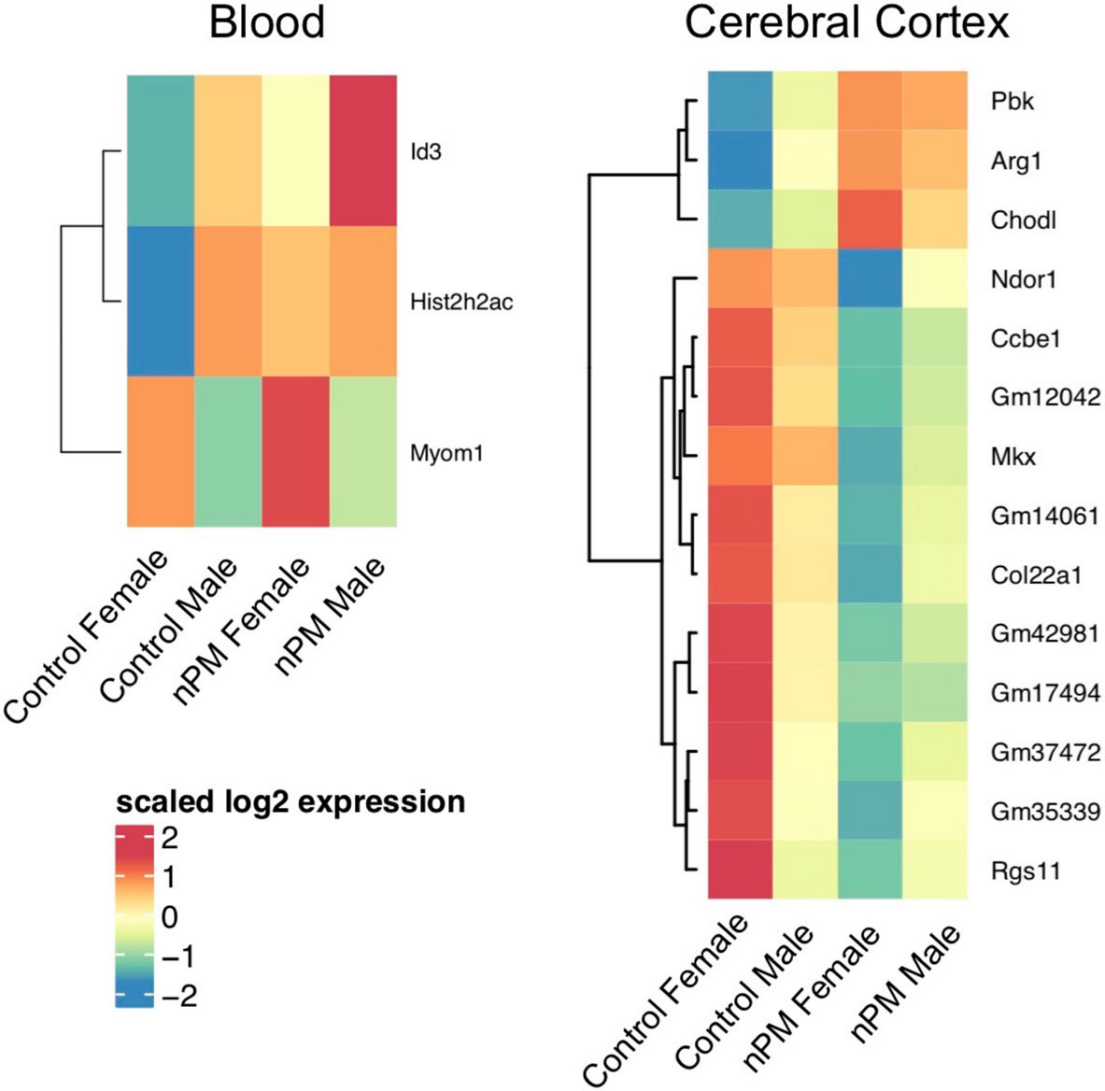
Sparse canonical correlation of nPM-associated gene in blood and cerebral cortex of mouse neonates. Heatmap showing the mean expression changes of the selected genes in blood and cerebral cortex. Gene expressions were adjusted for RIN values of each sample. These gene sets had a canonical correlation of 0.98

Comparison of the blood and cerebral cortex results revealed that nPM effects are mostly tissue specific. As shown above (Fig. 3), 2 nPM-DEGs were shared between blood and cerebral cortex. We further used a sparse canonical correlation analysis (CCA) of tissue-specific nPM-DEGs to select the blood nPM-DEGs that highly correlated with brain nPM-DEGs. This method will identify the blood nPM-DEGs that might not specifically change in the cerebral cortex, but correlate with the nPM-DEGs in the brain. Thus, the blood nPM-DEGs have the potential to be tested as biomarkers of air pollution mediated neurotoxicity. Our prior analysis identified a total of 382 nPM-DEGs in the cerebral cortex and 108 nPM-DEGs in blood. CCA of these subsets identified a group of 3 blood nPM-DEGs that canonically correlated (*r* = 0.98) with 14 nPM-DEGs in the cerebral cortex (Fig. 4). The blood genes included Id3 (inhibitor of DNA binding 3, involved in several pathways such as adipogenesis, Wnt, Hedgehog, and Notch), hist2h2ac (histone cluster 2, involved in meiosis and Rho GTPases), and Myom1 (Myomesin 1, involved in striated muscle contraction). Some example nPM-DEGs in the brain included Arg1 (arginase 1, associated with innate immune responses) and Col22a1 (collagen type XXII alpha 1).

## Discussion

This is a novel transcriptome analysis of cerebral cortex and blood of neonate mice with prenatal exposure to nPM. Prenatal nPM exposure induced 124 DEGs in the cerebral cortex of both sexes. Sex-specific effects were suggested with the greatest numbers of changes (322 DEGs) occurring in females. Simultaneous analysis of blood and cerebral cortex of the same animals identified 14 blood genes as potential biomarkers for the cerebral cortex gene expression changes: 2 nPM-DEGs were shared between blood and brain; and 3 nPM-DEGs were canonically correlated with brain responses. Some of the oxidative stress (e.g., NRF2) and immune responses were also shared between blood and cerebral cortex.

Prenatal nPM exposure affected 124 DEGs in the cerebral cortex that were related to immune system (e.g., neuroinflammation), brain development (e.g. axonal guidance), and some metabolism pathways. Other studies on developmental effects of PM also showed an increase of neuroinflammation [25, 34], microglial activation [29, 32], and neurodevelopmental changes such as ventriculomegaly and hypermyelination [34]. Our study highlighted the potential role of the Creb and Bdnf in the observed changes in the cerebral cortex. A recent study showed that prenatal PM2.5 exposure causes CREB/BDNF signaling activation in the hippocampus of one-month male and female neonates [52]. CREB is a transcriptional factor that regulates cell differentiation, proliferation, and survival in the nervous system. This gene is a target for cancer therapy through inhibition of phosphorylation, CREB-DNA, and CREB-CBP interactions [53]. The secreted neurotrophin brain-derived neurotrophic factor (BDNF) is a regulator of synaptogenesis, synaptic plasticity, neuronal differentiation, learning, and memory [54]. Further examination of the role of CREB and BDNF in air pollution toxicity mediated neurodevelopmental changes are warranted.

Several other enriched pathways of nPM-responsive genes in cerebral cortex of males and female neonates are supported experimentally based on our exploratory analyses. For example, in adult nPM-exposed male and female mice, selective cortical glutamatergic nPM effects are reported [41, 43, 55]. Another example is nitric oxide signaling and the potential regulatory role of nitric oxide synthase during nPM-mediated neurotoxicity. Our prior studies showed that nPM induces iNOS, nitric oxide, and/or nitrosylation in cell culture [56, 57], hippocampal slice [30], and in vivo [56]. Our analyses identified a subset of 322 nPM-DEGs in the female neonatal cerebral cortex after gestational exposure to nPM. Many epidemiological and mouse models document that prenatal air pollution exposure have sex-specific neurodevelopmental trajectories [30, 31, 33–36]. In humans, prenatal air pollution exposure affect the attention domains of boys and memory domains of girls [24]. Prior measurement of mitochondrial DNA copy number (mtDNAcn) as a marker of mitochondrial dysfunction in human cord blood and placenta showed off-spring sex-specific responses to gestational air pollution exposure [58, 59]. Mitochondrial dysfunction and oxidative phosphorylation were among the top nPM-responsive pathways in the cerebral cortex of both sexes of neonates, which parallels these studies. We believe replication and expansion of this sex-specific work in larger experimental samples may help to better understand mechanisms of sex-differences in a model system, with relevance to epidemiologic work.

Some of the potential upstream regulators of prenatal nPM:sex interaction included RICTOR, PSEN1, APP, MAPT (Tau) and NFE2L1 (NRF1). Our recent study in the adult brain revealed that sex can alter the antioxidant and neuroinflammatory responses to air pollution potentially through an intricate interplay of NRF2 and NFKB transcriptional factors [60]. The current study neuroimmune responses to air pollution in the neonates brain. In another study, we introduced Caenorhabditis elegans as a model of air pollution toxicity [61]. We showed initial nPM-mediated *skn-1*/*Nrf* homolog responses in the developmental stage can lead to long-term developmental and lifespan changes.This study also showed *sel-12*/*Psen* homolog is among the first larval stage nPM responses. This gene is a gamma secretase that is involved in amyloidogenesis and Alzheimer disease (AD). Exposure of adult AD mouse models to nPM lead to increase of cerebral cortex amyloid β levels [55]. The current results suggest gestational air pollution exposure might lead to sex-specific AD risk. Moreover, the identified upstream regulators can be used as preventive targets for the long-term depressive behavior, metabolic abnormalities, and potentially autism spectrum disorders observed at later ages [40].

Air pollution is a global risk factor of mortality and morbidity. The observed nPM-mediated gene expression profile suggest that prenatally exposed animals may be at a higher risk of mortality, morbidity, tumor formation, seizure, and learning deficits. A recent study estimated that high PM2.5 is responsible for 22% of infant death, around 449,000 death excess in more than 30 countries [1]. Cancer and neurodevelopmental effects of gestational air pollution are also documented in human studies [9, 62]. Potential hazards of gestational air pollution exposure on brain tumors warrant further epidemiological investigation.

Prenatal nPM exposure had fewer gene responses in the blood compared to the cerebral cortex. Despite the fewer number of changes identified, our results indicated some immune responses are shared between these two tissues. Thus, our study used mice transcriptome data to identify blood genes that can be tested in human studies as possible biomarkers of air pollution neurotoxicity. Studies show that gestational air pollution exposure leads to sex-specific telomere shortening [63, 64] and increase of PAH-DNA adducts in cord blood [2] but these have not been validated as potential biomarkers for brain effects. For the first time, we identified 5 blood genes (2 shared nPM-DEGs, 3 genes through CCA) associated with gene expression changes in the cerebral cortex of the offspring. The application of these genes in human cord blood or placenta should be tested in future studies.

There are still large gaps in knowledge regarding the mechanism of air pollution neurotoxicity. For example, it is unclear which neurotoxic components of PM contribute to the observed neurodevelopmental changes. Our recent study showed that the polycyclic aromatic hydrocarbons (PAHs) of PM are not required for some PM neurotoxicity [65], while prenatal exposure to PAHs is associated with neurodevelopmental and cognitive changes during childhood in epidemiologic data [21]. Another parameter not included in our study is maternal stress. Maternal resource deprivation interacts with gestational exposure to diesel exhaust particles (DEP) by inducing long-term offspring anxiety-like behavior and sex-specific gene expression changes; e.g., only male offspring with prenatal DEP and maternal stress showed increased Tlr4 and Casp1 [26]. Furthermore, in humans, a three-way interaction between PM2.5, maternal trauma and sex was shown for placental mitochondrial DNA copy number [58]. Future studies are needed to elucidate the contribution of maternal stress to air pollution-mediated transcriptome changes of neonates.

Several limitations of this study will require future studies to determine mechanisms by which nPM exposure changes gene expression and function in mouse brain and blood. Future studies will be required to validate transcript changes and protein abundance changes in a larger sample size. We did identify several robust differences in our analysis, but the small sample size limited the statistical power to identify smaller magnitude responses, in particular for sex-specific analyses. The single time point of analysis also limits our conclusions since we do not know how these transcriptional responses change during development. We also acknowledge that protein changes do not always correspond with transcriptional responses. Since proteins were not measured in our study, we cannot make definitive conclusions on functional outcomes potentially resulting from the observed transcriptional changes. Future studies need to include larger samples sizes, multiple time points, and protein measurements. The time course of neurodevelopmental transcriptional changes are particularly important because adult behavioral changes to nPM were found to be male-specific [40] while neonatal changes in gene expression were more pronounced in females.

## Conclusion

This novel study describes the transcriptome changes in cerebral cortex and blood of male and female neonates after prenatal air pollution exposure. Since we studied the changes in early postnatal stages, the observed changes can be applied as biomarkers or further studied as the biological responses that contribute to long-term effects of air pollution toxicity. We identified some blood genes that correlated with cortical responses to air pollution. Future analysis of these genes in human cord blood will determine their relevance.

## Supplementary Information

**Additional file 1: Supplementary file 1.** Differential expression analysis summary statistics.

## Data Availability

NCBI GEO Accession, GSE142453. Results in the supplementary excel file.
